# Supplementary material for: Type 2 diabetes disrupts circadian orchestration of lipid metabolism and membrane fluidity in human pancreatic islets
Source: PLoS Biol. 2022 Aug 3;20(8):e3001725. doi: 10.1371/journal.pbio.3001725 (PMC9348689; doi:10.1371/journal.pbio.3001725)
Supplement: S1 Fig — (DOCX) [file pbio.3001725.s001.docx]

***S1 Figure***

***S1 Fig.*** ***Oscillations of core-clock and clock-related genes in forskolin-synchronized human islets.***

RT-qPCR gene expression profiles of core clock genes *BMAL1, CLOCK, PER2, CRY2, REV-ERBα (REVA)*, and clock output genes *DBP* and *NFIL3* normalized to the mean of *9S* and *HPRT* in ND human islets synchronized *in vitro*. The data are expressed as mean ± SEM for n = 4 (n = 3 for *CRY2* and *NFIL3*) independent experiments, each using islets from one human donor. Note anti-phasic expression of *BMAL1* compared to the profiles of *PER2, REV-ERBα*, and *DBP*. See also S5 Data.
