# Supplementary material for: Type 2 diabetes disrupts circadian orchestration of lipid metabolism and membrane fluidity in human pancreatic islets
Source: PLoS Biol. 2022 Aug 3;20(8):e3001725. doi: 10.1371/journal.pbio.3001725 (PMC9348689; doi:10.1371/journal.pbio.3001725)
Supplement: S2 Fig — (DOCX) [file pbio.3001725.s002.docx]

***S2 Figure***


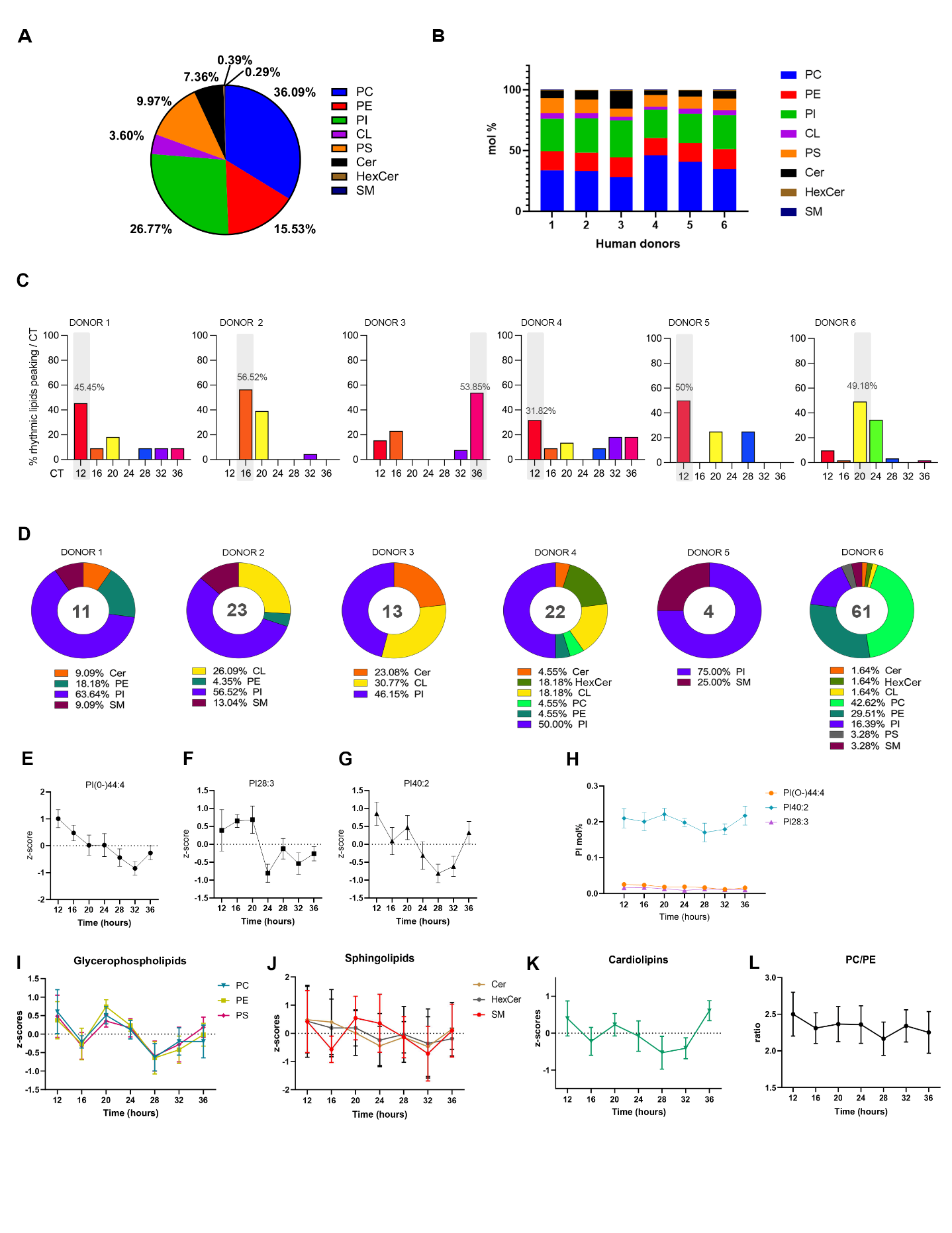


***S2 Fig. Lipid metabolites identified in human pancreatic islets from ND donors synchronized in vitro***

(A) Pie diagram illustrating the lipid class coverage of the lipids identified in human islets. Data represent the mean percentage of each lipid class across 7 time points and across the pancreatic islets from all ND human donors (n = 6).

(B) Relative abundance (mol %) of the different lipid classes in each islet extract used for the experiment depicted in Fig 1A.

(C) Graphs depicting the percentage of rhythmic lipids peaking at a certain collection point (CT) after synchronization with forskolin for each donor. The grey bar indicates the CT at which most rhythmic lipids peak and the color bars correspond to the different time points.

(D) Pie diagrams illustrating the lipid classes coverage of the rhythmic lipids identified for each donor. The percentage of rhythmic lipids clustered by lipid class is indicated below each pie. The average percentage of rhythmic lipids was 5.45 % across all islet donors based on percentage of circadian rhythmic lipids vs. detected lipids in each donor.

(E-G) Average PI(-O) 44:0 (J), PI28:3 (K), and PI40:2 (L) lipid profiles with lipid concentrations corrected for Class II isotopic over-laps.

(H) Abundance of the 3 PI rhythmic lipids identified and shown in Fig. 1E, as a percentage of total PIs detected at each time points.

(I) Average temporal profiles of glycerophospholipids abundance throughout the circadian cycle: PC, PE and PS species.

(J) Average temporal profiles of sphingolipids abundance throughout the circadian cycle: Cer, HexCer and SM species.

(K) Average temporal profiles of cardiolipins abundance throughout the circadian cycle.

(L) Average temporal profile of the ratio between PC and PE levels throughout the circadian cycle.

Phosphatidylcholine (PC), phosphatidylethanolamine (PE), phosphatidylinositol (PI), phosphatidylserine (PS), cardiolipin (CL), ceramide (Cer), hexosylceramide (HexCer), sphingomyelin (SM).

Data for (E-L) are represented as mean ± SEM, n = 6. See also S1 Data.
