# Supplementary material for: Type 2 diabetes disrupts circadian orchestration of lipid metabolism and membrane fluidity in human pancreatic islets
Source: PLoS Biol. 2022 Aug 3;20(8):e3001725. doi: 10.1371/journal.pbio.3001725 (PMC9348689; doi:10.1371/journal.pbio.3001725)
Supplement: S3 Fig — (DOCX) [file pbio.3001725.s003.docx]

***S3 Figure***


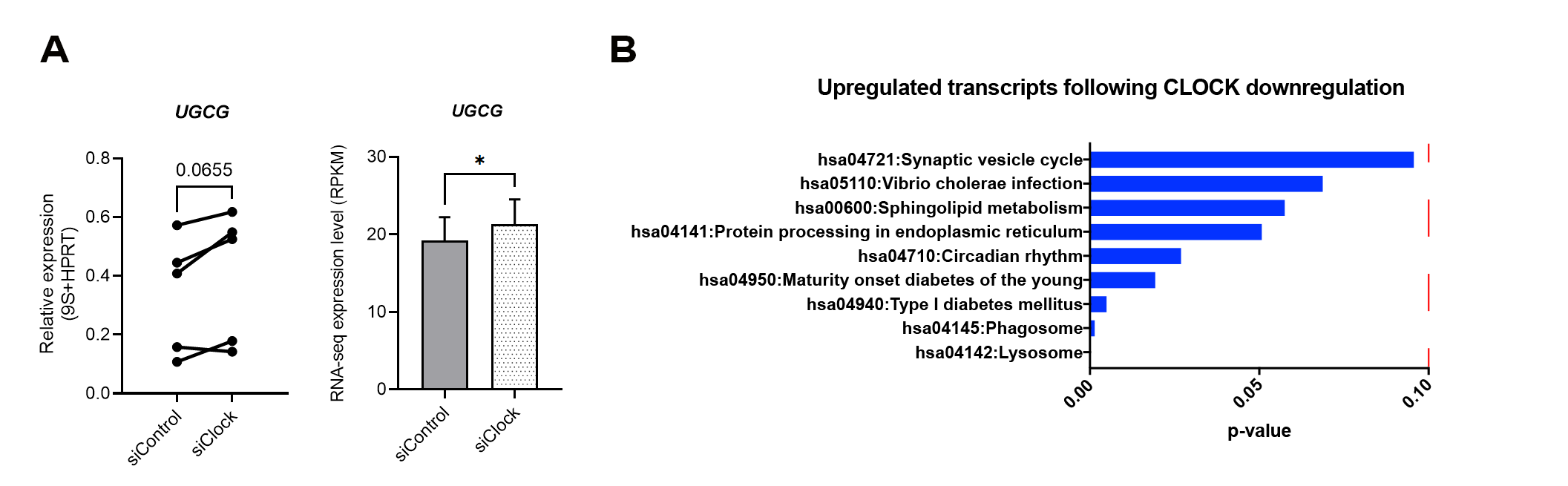


***S3 Fig.*** ***Upregulation of sphingolipid metabolism in clock-deficient human islet cells.***

(A) si*CLOCK*-mediated circadian clock disruption in ND human islet donors leads to upregulation of *UGCG* expression. RT-qPCR gene expression profile of UGCG normalized to HPRT and 9S expression in siControl and siClock transfected ND islets (left, n = 5, paired two-tailed t-test) and RPKM expression of UGCG measured by RNA-seq in ^1^ (n = 9, paired two-tailed t-test *P < 0.05).

(B) KEGG pathway analysis of upregulated transcripts following siRNA-mediated clock disruption in human islet cells (analyzed using DAVID online software, *p* < 0.1 based on the RNA-seq data from ^1^). The upregulated genes related to sphingolipid metabolism comprise *SGPL1*, *CERK*, and *NEU1*. See also S5 Data.

**References**

1 Saini, C. *et al.* A functional circadian clock is required for proper insulin secretion by human pancreatic islet cells. *Diabetes Obes Metab* **18**, 355-365, doi:10.1111/dom.12616 (2016).
