# Supplementary material for: Type 2 diabetes disrupts circadian orchestration of lipid metabolism and membrane fluidity in human pancreatic islets
Source: PLoS Biol. 2022 Aug 3;20(8):e3001725. doi: 10.1371/journal.pbio.3001725 (PMC9348689; doi:10.1371/journal.pbio.3001725)
Supplement: S4 Fig — (DOCX) [file pbio.3001725.s004.docx]

***S4 Figure***


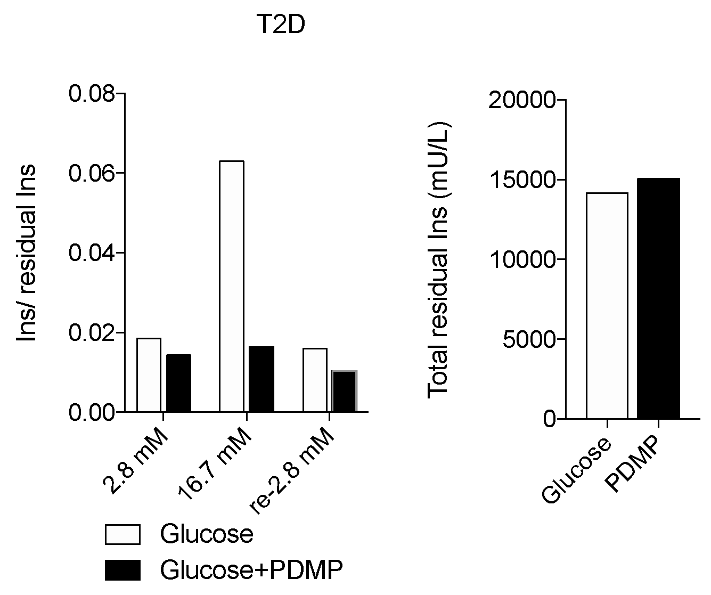


***S4 Fig. PDMP inhibits basal*** *(1-h at 2.8 mmol glucose)* ***and glucose-induced*** *(1-h at 16.7 mmol)* ***insulin secretion in human islet cells from T2D donor in vitro*** *(n = 1).* See also S5 Data.
