# Supplementary material for: Type 2 diabetes disrupts circadian orchestration of lipid metabolism and membrane fluidity in human pancreatic islets
Source: PLoS Biol. 2022 Aug 3;20(8):e3001725. doi: 10.1371/journal.pbio.3001725 (PMC9348689; doi:10.1371/journal.pbio.3001725)
Supplement: S5 Fig — (DOCX) [file pbio.3001725.s005.docx]

***S5 Figure***


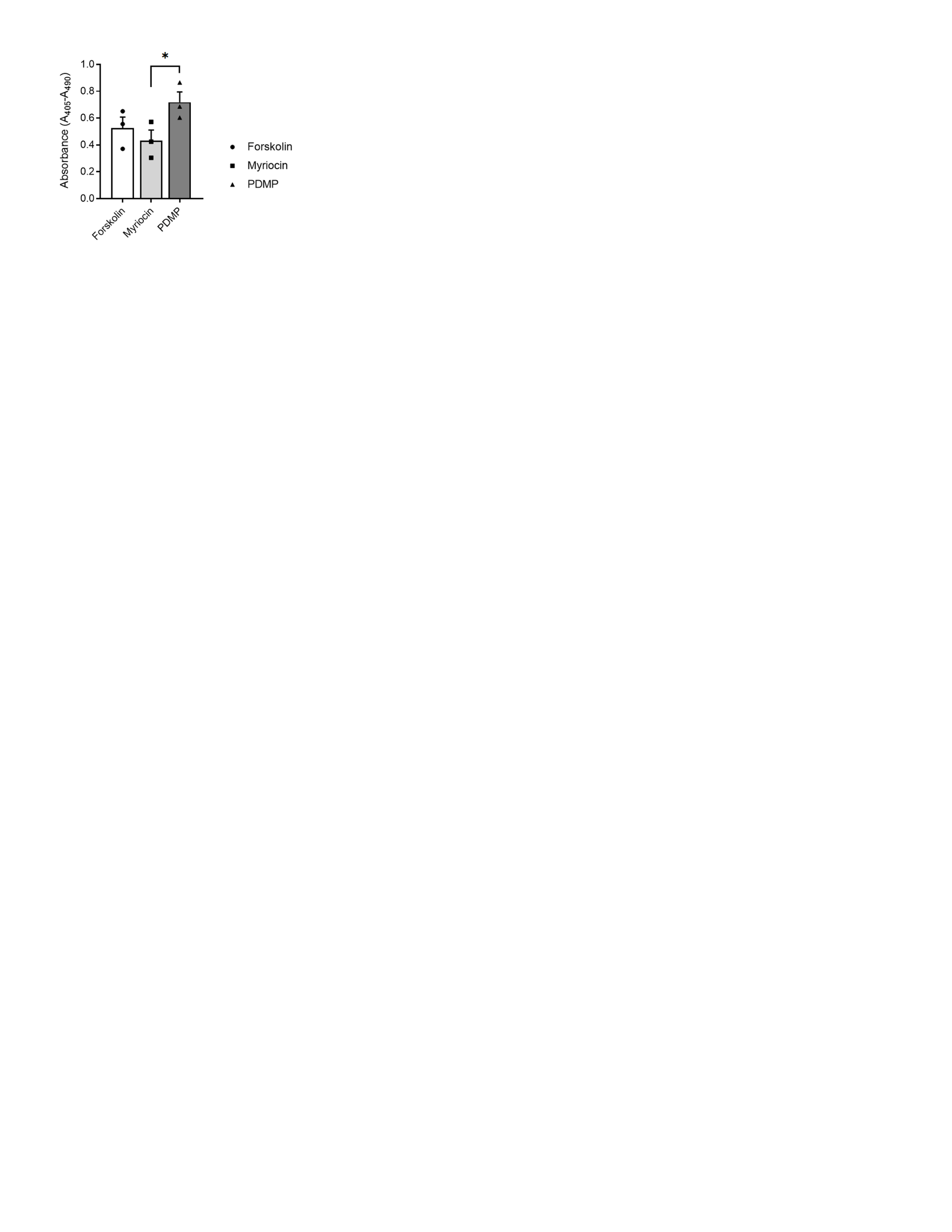


***S5 Fig.*** ***Assessment of apoptosis in pancreatic islets treated with myriocin or PDMP during continuous bioluminescence recordings for 5 days.***

The apoptosis measurement was conducted at the end of bioluminescence experiments for n = 3 ND donors. See also S5 Data.
