# Supplementary material for: Type 2 diabetes disrupts circadian orchestration of lipid metabolism and membrane fluidity in human pancreatic islets
Source: PLoS Biol. 2022 Aug 3;20(8):e3001725. doi: 10.1371/journal.pbio.3001725 (PMC9348689; doi:10.1371/journal.pbio.3001725)
Supplement: S1 Table — (DOCX) [file pbio.3001725.s006.docx]

***S1 Table. Sequences of quantitative RT-PCR primers***

| *Target gene* |  | *Sequence primers* |
| --- | --- | --- |
| *UGCG* | *forward* | 5’-AGACACCTGGGAGCTTGCTA-3’ |
|  | *reverse* | 5’-TTCGTCCTCTTCTTGGTGCT-3’ |
| *CERS2* | *forward* | 5’-TAGAGCTTTTGTCCCGGCAG -3’ |
|  | *reverse* | 5’-CGGCAATGAAGGCAATCAGG -3’ |
| *CEPT1* | *forward* | 5’-GGCTTGGGAAATCCTGTTAGC-3’ |
|  | *reverse* | 5’-TCTTTCCACTGAGTAAAACTGAGA-3’ |
| *CHKA* | *forward* | 5’-ACATCAGTGTCATCAGAGGCG-3’ |
|  | *reverse* | 5’- GAGCTTGTTCGGATCCCTCTT-3’ |
| *S9* | *forward* | 5’-CTCCGGAACAAACGTGAGGT-3’ |
|  | *reverse* | 5’-TCCAGCTTCATCTTGCCCTC-3’ |
| *HPRT* | *forward* | 5’-GATTTTATCAGACTGAGGAGC-3’ |
|  | *reverse* | 5’-TCCAGTTAAAGTTGAGAGATC-3’ |
| *BMAL1* | *forward* | 5’-CCCTTGGACCAAGGAAGTAGAA-3’ |
|  | *reverse* | 5’-CTTCCAGGACGTTGGCTAAAAC-3’ |
| *CLOCK* | *forward* | 5’-CCAGCCACCGCAACAATT-3’ |
|  | *reverse* | 5’-GGATTCCCATGGAGCAACCTA-3’ |
| *CRY2* | *forward* | 5’-ACTGCCCTGTGGGCTTTG -3’ |
|  | *reverse* | 5’-TGACATCCGGTTCGTCTACA -3’ |
| *PER2* | *forward* | 5’-CGCAGGGTGCGCTCGTTTGAA-3’ |
|  | *reverse* | 5’-GCTGGGCTCTGGAACGAAGCTTTCG-3’ |
| *DBP* | *forward* | 5’-TAGAAGGAGCGCCTTGAGTC-3’ |
|  | *reverse* | 5’-GCAACCCTCCAGTATCCAGA-3’ |
| *REV-ERBα* | *forward* | 5’-GATCGTGAGTCGCGGGGTCC-3’ |
|  | *reverse* | 5’-TGTAGGTGATGACGCCACCTGTGT-3’ |
| *NFIL3* | *forward* | 5’-GGAGAAGACGAGCAACAGGT -3’ |
|  | *reverse* | 5’-CTTTGATCTGCATGGCTTTG -3’ |
