# Supplementary material for: Type 2 diabetes disrupts circadian orchestration of lipid metabolism and membrane fluidity in human pancreatic islets
Source: PLoS Biol. 2022 Aug 3;20(8):e3001725. doi: 10.1371/journal.pbio.3001725 (PMC9348689; doi:10.1371/journal.pbio.3001725)
Supplement: Attachment — Submitted filename: Response_to_Reviewers.docx [file pbio.3001725.s012.docx]

**Point by point response to the comments by the Editorial Board and the Referees**

**Petrenko et al., PBIOLOGY-D-22-00209R1**

Dear Dr Dibner,

Thank you for submitting your manuscript entitled "Circadian orchestration of lipid metabolism and membrane fluidity in human pancreatic islets are disrupted upon type 2 diabetes" for consideration as a Research Article at PLOS Biology. Thank you also for your patience as we completed our editorial process, and please accept my apologies for the delay in providing you with our decision. Your manuscript has been evaluated by the PLOS Biology editors, an Academic Editor with relevant expertise, and by three independent reviewers.
You will see that the reviewers find the conclusions novel and interesting, however they each raise several points that should be clarified or amended to strengthen the results of the manuscript. Reviewers 1 and 2 ask mainly for clarifications, whereas Reviewer 3 raises two points that require the generation of additional data, but we think they can be easily addressed.
In light of the reviews (attached below), we are pleased to offer you the opportunity to address the comments from the reviewers in a revised version that we anticipate should not take you very long. We will then assess your revised manuscript and your response to the reviewers' comments and we may consult the reviewers again.
We expect to receive your revised manuscript within 1 month.
Please email us (plosbiology@plos.org) if you have any questions or concerns, or would like to request an extension. At this stage, your manuscript remains formally under active consideration at our journal; please notify us by email if you do not intend to submit a revision so that we may end consideration of the manuscript at PLOS Biology.

**IMPORTANT - SUBMITTING YOUR REVISION**
Your revisions should address the specific points made by each reviewer. Please submit the following files along with your revised manuscript:

1. A 'Response to Reviewers' file - this should detail your responses to the editorial requests, present a point-by-point response to all of the reviewers' comments, and indicate the changes made to the manuscript.
*NOTE: In your point by point response to the reviewers, please provide the full context of each review. Do not selectively quote paragraphs or sentences to reply to. The entire set of reviewer comments should be present in full and each specific point should be responded to individually, point by point.
You should also cite any additional relevant literature that has been published since the original submission and mention any additional citations in your response.

2. In addition to a clean copy of the manuscript, please also upload a 'track-changes' version of your manuscript that specifies the edits made. This should be uploaded as a "Related" file type.

3. Resubmission Checklist
When you are ready to resubmit your revised manuscript, please refer to this resubmission checklist: https://plos.io/Biology_Checklist

To submit a revised version of your manuscript, please go to https://www.editorialmanager.com/pbiology/ and log in as an Author. Click the link labelled 'Submissions Needing Revision' where you will find your submission record.

Please make sure to read the following important policies and guidelines while preparing your revision and fulfil the editorial requests:

a) *PLOS Data Policy*
Please note that as a condition of publication PLOS' data policy (http://journals.plos.org/plosbiology/s/data-availability) requires that you make available all data used to draw the conclusions arrived at in your manuscript. If you have not already done so, you must include any data used in your manuscript either in appropriate repositories, within the body of the manuscript, or as supporting information (N.B. this includes any numerical values that were used to generate graphs, histograms etc.). Please also indicate in each figure legend where the data can be found. For an example see here: http://www.plosbiology.org/article/info%3Adoi%2F10.1371%2Fjournal.pbio.1001908#s5

b) *Published Peer Review*
Please note while forming your response, if your article is accepted, you may have the opportunity to make the peer review history publicly available. The record will include editor decision letters (with reviews) and your responses to reviewer comments. If eligible, we will contact you to opt in or out. Please see here for more details:
https://blogs.plos.org/plos/2019/05/plos-journals-now-open-for-published-peer-review/

c) *Blot and Gel Data Policy*
Please provide the original, uncropped and minimally adjusted images supporting all blot and gel results reported in an article's figures or Supporting Information files. We will require these files before a manuscript can be accepted so please prepare them now, if you have not already uploaded them. Please carefully read our guidelines for how to prepare and upload this data: https://journals.plos.org/plosbiology/s/figures#loc-blot-and-gel-reporting-requirements

d) *Blurb*
Please also provide a blurb which (if accepted) will be included in our weekly and monthly Electronic Table of Contents, sent out to readers of PLOS Biology, and may be used to promote your article in social media. The blurb should be about 30-40 words long and is subject to editorial changes. It should, without exaggeration, entice people to read your manuscript. It should not be redundant with the title and should not contain acronyms or abbreviations. For examples, view our author guidelines: https://journals.plos.org/plosbiology/s/revising-your-manuscript#loc-blurb

e) *Protocols deposition*
To enhance the reproducibility of your results, we recommend that if applicable you deposit your laboratory protocols in protocols.io, where a protocol can be assigned its own identifier (DOI) such that it can be cited independently in the future. For instructions see: https://journals.plos.org/plosbiology/s/submission-guidelines#loc-materials-and-methods

Thank you again for your submission to our journal. We hope that our editorial process has been constructive thus far, and we welcome your feedback at any time. Please don't hesitate to contact us if you have any questions or comments.

Sincerely,
Ines
--
Ines Alvarez-Garcia, PhD
Senior Editor
PLOS Biology
ialvarez-garcia@plos.org
------------------------------------------------------------------

Reviewers' comments

Rev. 1:
In this manuscript, Petrenko et al examine the lipidome of pancreatic islet cells from normal donors (ND) and type-II diabetes (T2D) subjects. They demonstrate a number of lipid species and classes are oscillatory ex-vivo in isolated cells from NDs, as well as alterations in glycerophospholipids (GPLs) and sphingolipids in T2Ds between two timepoints. A strength of the study is the further investigation of these lipid alterations by examination of key enzymes of sphingolipid regulation which showed rhythmicity in NDs, and disruption by clock perturbation in NDs as well as in T2Ds. T2Ds also demonstrated reduced membrane fluidity similar to clock-disrupted NDs. Finally the authors demonstrate reduced glucose induced insulin secretion from inhibition of glycosphingolipid synthesis, while inhibiting earlier step ceramide synthesis elicits a weaker response. The authors concluded that that the circadian clock is required for temporal control of lipid homeostasis and disruption of the clock impairs insulin secretion.

Overall the work is clearly written and easy to follow, and the experimental approach is logical. Unfortunately the quality of the figures in the pdf I had was quite low and the figures were difficult to evaluate.

The authors thank the Referee for the very constructive and complimentary evaluation. Please find our point-by-point responses below.

A few limitations or clarifications which should be discussed:

1. The lipidomics experiments were performed on cells from six NDs, however these individuals span a dramatic range in BMI (23.7-44.1). Given the fairly small number of NDs in this experiment, this has an important impacts on the overall lipid cycling and the ability to detect cycling lipids. As the authors note from the Chua et al, individual lipid profiles in blood demonstrate immense variability in cycling. While the approach to select for lipids cycling in 3/6 NDs mitigates this to some extent, the combination of low numbers and heterogenous population is likely hampering discovery of potentially important cycling lipids and / or classes. Was other clinical chemistry information available from these donors such as TGs or other lipid panels? In summary, the total number of cycling lipids is probably vastly underestimated.

We entirely agree with this fair point raised by the Referee. Unfortunately, we have access to a limited number of clinical characteristics and blood biochemistry parameters of the islet donors, such as blood group and antibodies to CMV, HIV, HBsAg, HCV, HTLV, EBV, anti-HBc. However, no additional information regarding TGs or other lipid measurements, that would have indeed been very helpful for detailed individual analysis of the lipidomics data, has been available. All the available clinical information related to the human islet donors is listed in the Table 1.

We entirely agree with the Reviewer that circadian rhythms of lipid metabolites are highly variable across islet batches isolated from different human donors, as has been previously demonstrated by Chua and colleagues for the circadian analyses of human serum samples ^1^, **and in our own lipidomic study of human skeletal muscle across 24 h ^2^. Concordantly, we indeed observe high variability in the rhythmic profiles of lipid metabolites across the subjects, as can be seen in Fig 1 and revised Fig S2, resulting in** substantial inter-individual differences in the number of rhythmic lipids, and in distinct phase and amplitude of lipid metabolite rhythms. As pointed out by the Referee, due to the overall low number of subjects utilized for these elaborate analyses, sub-clustering across the subjects makes very little sense in this study, as it was done in above cited works where 10 or more human participants were enrolled allowing such sub-clustering ^1,2^. We entirely agree with the Referee that 5 % or rhythmic lipid metabolites that we report is likely to be an underestimation, due to such low number of islet donors, largely distinct donor characteristics (sex, BMI, etc.), in addition to differences in islet preparation quality and intrinsic heterogeneity of pancreatic islets. Following the constructive remark by the Referee, we further developed this important point in the Discussion chapter as following:

“In our previous lipidomic analysis we report that in synchronized human primary myotubes the circadian oscillating lipid species were more abundant, reaching up to 18.6% **^28^**. Such discrepancy may reflect tissue-specific lipid composition, or stem from the high inter-donor variability, low number of the islet donors (n = 6), limited amount of starting material, and islet cellular heterogeneity. »

2. Some explanation for why the ND cells used for lipidomics were not the same as those used for the qPCR of enzyme analysis is required. Given the concerns above, this is particularly important as there is no guarantee that the inter-individual variability does not extend to these enzymes (and hence a potential mismatch cycling between the lipids and enzymes). In general, the use of different donors for the different aspects of the study is a weakness (although I understand that acquiring sufficient material may be an issue).

We thank the Referee for pointing this out, and entirely agree with this criticism. Indeed, one of the major limitations of the research conducted in human islets isolated from deceased donors is lack of material. In practical terms, to perform the lipidomics analyses around-the-clock in 7 time-points in duplicate dishes with 600 islets per dish that underly Fig 1, a minimum of 8400 human islet equivalents (IEQ) are required per experiment. In few cases, namely for the donors ND 5 and T2D 3-4, we received enough IEQ to conduct lipidomics along with functional studies and qRT-PCR analyses. However, for the rest of the donors we could only perform lipidomics based on the IEQ number obtained in each batch. Therefore, qRT-PCR analyses had to be conducted based on the islet material from different donors. We added this important clarification to the Discussion chapter as following:

“These temporal variations are correlated with the rhythmic transcription of *CERS2* and *UGCG*, the key enzymes involved in ceramide and glucosylceramide synthesis (Fig 4A, C), as well as with the expression of core clock transcripts (Fig S1). Of note, in some instances mRNA measurements were conducted on the islets derived from different donors from those utilized for the lipidomics analyses (see Table 1).”

3. Why were 12 and 24 h chosen as the time points for the T2D lipidomics analysis? There are a range of peaks phases apparent in Supp Fig 1C, and while a number peak at 12, these appear to be different lipid classes for each donor (assuming the colors relate to the classes in Supp Fig 1B; its not made clear). I think some aggregate analysis of phase properties would be appropriate to include in Figure 1. In addition, some discussion of those lipids / lipid classes that peak between ZT 6 or 18 is worth mentioning as these are likely not going to be capture in the current design.

We thank the Referee for this pertinent and constructive criticism. We entirely agree that experimental design based on 2 time points 12 h apart from each other does not allow recapitulating the lipid rhythmicity, in particular for the metabolites that do not peak at CT12/24. Two time points instead of usual 7 was a compromise that had to be made due to the lack of T2D islet material that is even more important than for ND islets (see the response to previous comment 2). Based on our available sources of human islets, we simply cannot obtain enough IEQ from T2D donors for the full-blown around-the-clock lipidomic design as was done for the experiments in ND islets presented in Figure 1. It is clear that any 2-time point design would lead to missing part of the rhythmic lipid metabolites, and all metabolites that peak at CT6/18 will not be qualified as changing across 24 h in our study design.

The opposite time-points 12 h and 24 h of the oscillatory cycle were chosen based on the transcriptional data, and on the oscillatory profiles of most of the lipids in ND donors. Indeed, these 2 points correspond to peak and trough of the core-clock gene *BMAL1* expression level (revised Fig S1), and of the rhythmic profiles obtained for lipid species in most of the examined ND donors (4 out of 6 donors, Fig 1C).

“There are a range of peaks phases apparent in Supp Fig 1C, and while a number peak at 12, these appear to be different lipid classes for each donor (assuming the colors relate to the classes in Supp Fig 1B; its not made clear). “

We thank the Referee for pointing out the lack of clarity regarding the color code used for different figure panels. Of note, the color codes on the original Fig S1C (revised Fig S2C) do not correspond to the color codes on the original Fig S1B (revised Fig S2B). In the Fig S1C (revised Fig S2C), the different colors correspond to the different time points, and not to different lipid classes. We now added this precision to the legend of the revised Fig S2C, to clarify the color code significance. Furthermore, the Figure S1D (revised Fig S2D) illustrates the lipid classes coverage of the rhythmic lipids identified for each donor.

“I think some aggregate analysis of phase properties would be appropriate to include in Figure 1.”

We entirely agree with the Referee regarding the pertinence of aggregate analysis of the lipid circadian phases. Indeed, we initially conducted phase clustering analyses of all lipid metabolites prior to data filtering for assessment of the circadian rhythms that yielded important number of rhythmic lipid species. This analysis is presented below in Figure 1 Referee 1. However, when filtering was applied to sort out the lipid metabolites that have very low abundance and thus risk to be misidentified by the MS, only few lipid metabolites were qualified as circadian rhythmic. Their phase distribution is presented in revised Fig S2C of the manuscript. We thus believe that more oscillating lipid species would be required in order to present the proposed phase aggregation that indeed could be very informative. With our final dataset of circadian rhythmic lipid species, it seems to us difficult to come up with a more advanced phase clustering than the one presented in manuscript in revised Fig. S2C.

**Figure 1 Referee 1. Phase aggregation analyses of the lipid classes in 6 non-diabetic donors (related to Fig. S2C of revised manuscript).** Phase distribution according to the lipid classes was done using k-means clustering.

Following constructive criticism by the Referee, we underscored this important limitation of our study design in T2D islets in the following remarks to the revised text of Results and Discussion:

Results

“The selected time-points correspond to peak and trough of the core-clock gene *BMAL1* expression level (Fig S1), and of the rhythmic profiles obtained for lipid species in most of the examined ND donors (4 out of 6 donors, Fig 1C).”

Discussion

“Due to the limited availability of human islets derived from T2D donors, we were unable to perform complete around the clock experiments for this part and compromised on 2-timepoint design. As a result, the temporal changes in lipid metabolites that are peaking at CT6 and CT18 were likely missed in T2D islets.”

Furthermore, we toned down the message on temporal analyses in T2D islet in the respective sub-chapter in Results, since it is indeed an overstatement, and reformulated it as following:

“Lipidomic profiling reveals major changes in lipid metabolites at 2 time points in human T2D pancreatic islets synchronized *in vitro”*

4. Lines 255-258: The authors suggest that the increase in UGCG mRNA is indicative of glucosylceramide production as opposed to galactosylceramides due to a simarity in profile. Other circadian studies have suggested that the lag between mRNA expression and protein expression averages to ~6 h (Mauvoisin et al, 2014; Robles et al, 2014) and that in certain pathways the lag to metabolites is ~12 h (Krishnaiah et al, 2017). While there is wide variance in this literature data, the temporal nature of protein expression and function does not support the claim being made in the current manuscript in my opinion. A similar association is suggested on lines 262-269 for Ceramide synthase 2.

We thank the Referee for this criticism. Indeed, there is a lag between mRNA and protein expression that largely varies for each protein translation rate. Since indeed we did not measure RNA/protein dynamics for UGCG and CERS2 around the clock our claim is an overstatement. We toned it down in the revised Results chapter, and it now reads as following:

“Strikingly, the *UGCG* mRNA expression measured around the clock in the islets from ND donors exhibited a rhythmic profile (p = 0.05 as assessed by JTK_Cycle) with the trough around 20-24h following *in vitro* synchronization, corroborating the temporal profile of *BMAL1* transcript (Fig S1), and recapitulating the diurnal accumulation profile of HexCer (Fig 4A).”

5. The authors use PDMP to inhibit UCGC and demonstrate a significant impact on glucose induced insulin secretion. PDMP has been shown to have other effects on cells in addition to impacting glycolipid synthesis, including alterations on membrane structure and cholesterol homeostasis. The potential glycophosolipid-independent effects of PDMP on GSIS should be discussed.

Indeed, in addition to glucosylceramide synthase inhibition, PDMP was shown to attenuate mTOR signaling pathway and to induce lysosomal lipid accumulation ^3^. We entirely agree that these effects may interfere with insulin release and glucose sensing, and thus play a role in observed inhibition of insulin secretion. Our new experiments conducted during the revision period indicate that application of PDMP during triggering glucose-independent depolarization of human islet cells with 30 mmol KCL for 1-h inhibits insulin secretion (new Fig 6E). These data suggest that effect of PDMP rather relies on granule exocytosis, and not on insulin sensing. Additional study, that employed direct downregulation of UGCG via siRNA in mouse islets, and that bypasses potential off-target effects of PDMD, also resulted in major inhibition of insulin secretion ^4^, further suggesting specific role of this enzyme in insulin exocytosis.

These points are now summarized in the Discussion as following:

“In addition to glucosylceramide synthase inhibition, the attenuation of mTOR signaling pathway and lysosomal lipid accumulation reported following PDMP treatment ^76^ may partly account for its inhibitory effect on insulin secretion. Importantly, knock-down of *UGCG* by siRNA in mouse islets also resulted in major insulin secretion defects ^77^, further suggesting that UGCG plays a key role in the observed phenotype.”

Other comments:

6. The numbers of donors for each experiment should be specified in the abstract for clarity.

The authors thank the reviewer for this suggestion. The number of donors used for the temporal profiling was now added to the abstract for each study group.


Rev. 2:
In this manuscript by Petrenko et al, the authors explore the circadian control of lipid species and membrane fluidity in ND and T2D islets. The authors demonstrate that lipid species profiles vary on a daily basis and that these profiles are altered in T2D islets. They further demonstrate that membrane fluidity is altered in both T2D islets and in islets where CLOCK has been knocked down. Additionally, it is demonstrated that disruption of de novo ceramide synthesis and glycosphingolipid biosynthesis impair insulin secretion and that ceramide turnover feeds back on circadian oscillations in the islets. Overall, the authors conclude that a functional circadian clock is required for the temporal control of lipid homeostasis and that disruption of lipid metabolism in T2D contributes to altered membrane fluidity and ultimately impaired GSIS.

This is a well-written and clearly presented manuscript. The data is convincing, the approaches are clearly described and the conclusions drawn are supported by the data presented. This reviewer only has minor comments/suggestions to provide a few points of clarity.

The authors thank the Referee for the constructive and complimentary commentaries, and for a positive evaluation of our piece.

1. In table 1, the term "Gender" is used in the second column. It appears the authors meant to refer to biological sex as genders are not actually provided in this table. That said, this column should be changed to "sex" and notably, Male (M) and Female (F) are the typical terms used, not M and W

Following constructive suggestion by the Referee, we corrected the wording in the revised Table 1 accordingly.

2. Line 178 - this subtitle wording is awkward. Suggest something like: "Lipidomic profiling reveals major changes in lipid metabolites at two time points in synchronized human T2D islets"

We are grateful to the Referee for this constructive criticism. Indeed, the original sub-title was an over-statement given that the profiling was only conducted in 2 time points. We modified the sub-title as suggested, and it now reads as:

“Lipidomic profiling reveals major changes in lipid metabolites at 2 time points in human T2D pancreatic islets synchronized *in vitro*”.

3. Line 181: Should be "to measure" not measuring

The authors thank the Reviewer for pointing out this error. The phrase has now been corrected as following:

“After identifying circadian rhythmic lipid metabolites in the pancreatic islets from ND donors cultured and synchronized *in vitro*, we next attempted to measure their temporal alterations in T2D islets.”

4. While this reviewer recognizes that journals have limits to the number of figures, there are many figures in the supplemental figures that should be considered for use in the primary figures. For example, on page 8 in the results section, the entire first paragraph on the lipidomic profiling section refers entirely to supplemental data. I would suggest that the authors reconsider the data in the supplemental figures and when there are significant differences that are discussed in the results, these figures should be presented in the primary figures.

Indeed, the space limitation is an issue that prevented from us including considerable amount of data to the main figures in the original manuscript. We agree that we extensively presented the data included in supplementary figure panels, in particular the data presented in Fig S3 from the original manuscript (p. 8). We have now introduced Fig S3 as main Figure (revised Fig. 2). To meet the space limitations with regard to the number of main figures, we fused original Fig 5 and 6 into Revised Fig 6. The legends of revised Fig 2 and revised Fig 6 were re-written accordingly, and all the changes were introduced to revised main text.

5. Results line 206-208: "Consistently, we noticed a higher level of total hexosylceramides in the T2D group compared to the ND group at both time points…". This is not obvious in the graph presented in 2H. The authors should consider including an inset that only has this condition on a smaller scale so that the difference can be better visualized.

Indeed, the scale of the graph at the original panel 2H (revised 3H), presenting all the lipid class levels, does not allow to distinguish the difference between the 2 groups. To highlight this difference, we zoomed into the HexCer levels only. Thus, revised panel 3I (original 2I) allows to demonstrate a significant increase of HexCer levels in the T2D group in 2 time points, 12h and 24h following *in vitro* synchronization. The difference is also visible in the panels of revised Fig 2J-K (original Fig. S3), where we presented the levels of DHCer, Cer, HexDHCer and HexCer in islets from T2D and ND at both time points. We modified the citation of these figure panels in the main text in order to refer more accurately to the panels that support our statement:

“Consistently, looking at the levels of all major lipid classes (Fig 3G-H), we noticed a higher level of total HexCer in the T2D group compared to the ND group at both time points with marked difference at 24 h (Fig 3I; Fig 2J-L).”

6. Figure 2K, L: It would be helpful to include PC/PE ratio on the x-axis rather than just stating ratio.

As suggested by the Referee, we changed the title of y-axis for the two graphs presented in Revised Figure 3K-L (original Fig. 2K-L).

7. Results line 218-219: "…all unsaturated PE subspecies were decreased in the T2D group, regardless of their degree of saturation (Fig 2M-N)". This statement doesn't seem fully supported by the data in figure 2N as PUFA4 and 5-6 don't seem to show a significant reduction in T2D, as presented in the figure. Consider re-wording this statement.

We thank the Referee for this constructive commentary. Indeed, although the decrease of unsaturated PE species in the T2D group was observed across all the lipids irrespective of their degree of saturation, it was not statistically significant for PUFA4 (at 12h and 24h) and for PUFA5-6 (at 24h).

To tone-down the message, we added the following precision:

“Whereas all unsaturated PE subspecies, regardless of their degree of saturation, exhibited the trend toward the decrease in the T2D group, this difference did not reach significance for PUFA 4 (at both time points) and PUFA 5-6 (at 24 h) (Fig 3M-N). In contrast, this difference was highly significant for PUFA 3, even after deisotoping correction of the lipid signals (Fig 2P-Q).”

8. Subtitle on line 249-250: This subtitle is a little awkward. Suggest removing the word "for" from the subtitle.

The authors thank the Referee for this correction. We changed the subtitle accordingly:
«Diurnal ceramide levels correlate with transcript profiles encoding key enzymes involved in their turnover»

9. For qPCR data in Figure 3 - how was the data normalized? It is understood from the materials and methods that the data is expressed relative to a housekeeping gene, but was this data at 24 hours expressed relative to the data at 12 hours in each condition?

Through the manuscript, the expression of genes of interest measured by qRT-PCR was normalized to average expression of 2 housekeeping genes (9S and HPRT). This precision was now added to the y-axis in revised Fig. 4, to the newly added Fig S1, and to revised Fig S3. Additionally, it is highlighted in the Material and methods section as following:

“Mean values for each sample were calculated from technical duplicates of each quantitative RT-PCR (qRT-PCR) analysis and normalized to the mean of housekeeping genes *hypoxanthine-guanine phosphoribosyltransferase* (*HPRT*) and *S9*. Primers used for this study are listed in Table S1.”

10. Discussion line 463: "…myriocin dampened the levels of secreted insulin in both ND and T2D human islet cells". This statement seems to be a bit of an over statement as myriocin did not significantly impair GSIS at 16.7mM only insulin secretion at 2.8mM was significantly affected.

We entirely agree with this criticism raised by the Referee. Indeed, myriocin exhibited statistically significant inhibitory effect on basal insulin secretion only (revised Fig 6A-C). We toned down this conclusion, and changed the sentence in the Discussion as following:

“Here we show that *de novo* production of sphingolipids is required for normal secretion of insulin by human islet cells *in vitro*, since application of sphingolipid synthesis inhibitor myriocin dampened the basal levels of secreted insulin in both ND and T2D human islet cells (Fig 6A-C).”

11. Discussion Lines 466-480: How do you reconcile the increased apoptosis in the PDMP treatment condition? This should also be discussed and considered in the interpretation of the "impaired" GSIS results.

Indeed, the experimental design underlying the results demonstrated in revised Fig 6F, G relies on the continuous recording of circadian bioluminescence from synchronized pancreatic islets cells for 5 days, in the absence or presence of PDMD or myriocin. Revised Fig S5 represents the level of apoptosis measured in the same pancreatic islets in the end of the recording at the same 3 conditions (control, PDMD, myriocin). Importantly, the statistical significance was only reached when myriocin - treated islets were compared to those treated with PDMD. By contrast, comparison of the cell death levels between either PDMD and untreated control islets, or myriocin and untreated control, did not yield significant difference.

In addition to glucosylceramide synthase inhibition, PDMP was shown to attenuate mTOR signaling pathway and to induce lysosomal lipid accumulation ^3^ that may potentially be involved in triggering the apoptosis. These effects may indeed potentially interfere with insulin release and glucose sensing, and thus play a role in observed inhibition of insulin secretion.

Our new revision experiments show that short-term application of PDMP during glucose-independent depolarization of human islet cells with 30 mmol KCL for 1-h also resulted in a strong inhibition of insulin secretion (revised Fig 6E), suggesting that the effect of PDMP rather relies on granule exocytosis, and not on insulin sensing. Although we did not measure the apoptosis level after acute exposure to PDMP that we had during GSIS experiments, beta-cell apoptosis would lead to uncontrolled release of intracellular insulin content resulting rather in enhanced insulin secretion, and not in its inhibition.

To summarize, we do not believe that short exposure to PDMP that is used for GSIS and KCl stimulation experiments shown in revised Fig 6 (A-E) leads to significant levels of islet cell apoptosis. Moreover, the 5-day exposure to the PDMD that was used for bioluminescence experiments (revised Fig 6 F-G) led to a trend toward increase in cell apoptosis that did not reach statistical significance (Fig S5, compare forskolin alone and forskolin plus PDMP). We therefore think that the weak proapoptotic effect of PDMD observed in long term experiments has no or very little impact on the acute settings used in GSIS.

Rev. 3:

In this manuscript Petrenko and colleagues follow up on a previous body of work detailing transcriptional and functional properties of human islets that are subject to circadian clock control in vitro by attempting to mechanistically probe the function of rhythmic phospholipid and sphingolipid metabolism in glucose-stimulated insulin secretion. They apply lipidomic analysis to non-diabetic, type 2 diabetic and siCLOCK treated human islets and show that specific subsets of phospholipids and ceramide metabolite precursors of sphingolipids accumulate rhythmically and are altered by CLOCK silencing and diabetes. They link clock transcription-factor mediated control of lipids perturbed in diabetic islets to islet function by demonstrating that perturbing glucosylceramide synthesis (by increasing UGCG transcript) inhibits insulin secretion, possibly by altering the disposal of toxic ceramide species or influencing membrane fluidity. Importantly, this manuscript elaborates a layer of circadian control occurring at the level of lipid homeostasis in a key glucose-regulatory tissue that has been understudied, particularly in primary human cells. I think the work presented here is novel and will benefit and may facilitate further intellectual exchange between the circadian, diabetes and metabolic physiology research communities. However, I have several suggestions to strengthen the manuscript:

Major points:

1. The authors demonstrate that ceramides and genes involved in ceramide and sphingolipid metabolism are rhythmic and increase in the setting of CLOCK inhibition or diabetes, however there are a number of possibly addressable missing links between clock transcription factors and lipid metabolism-related enzymes. First, it would help if the authors would superimpose or compare the RNA expression profiles of transcriptional activators (CLOCK, BMAL1, DBP) and repressors (PERs, CRYs, REVERB, E4BP4) with key lipid-biosynthetic enzymes pointed out throughout the manuscript. This would help in understanding how the circadian clock network is regulating lipid composition. Next, the authors should compare the expression of CLOCK and other components of the clock transcription loop in their T2D and siCLOCK samples to determine the extent to which changes in expression of clock transcription regulators might underlie lipid changes in the T2D samples. Lastly, given that many of the ceramide synthesis genes were up-regulated in T2D and UGCG transcript is increased in siCLOCK islets it would be useful to determine if any known circadian repressors are up-regulated in these settings and/or if canonical circadian repressor motifs are present in the promoters of these genes.

We are grateful to the Referee for raising this pertinent point. Following this constructive suggestion, we measured transcript levels of core-clock and clock-regulated transcripts from the same RNA samples extracted from the pancreatic islets derived from ND donors collected around-the-clock that were utilized for the assessment of lipid-biosynthesis enzymes (Fig. 1 Referee 3; new Fig S1). The results indicate that the temporal expression profiles of *UGCG* and *CERS2* exhibited a rhythmic profile (p = 0.05 as assessed by JTK_Cycle) with the trough around 20-24h following *in vitro* synchronization, corroborating the temporal profile of *BMAL1* transcript (New Fig S1; Fig. 1 Referee 3), and recapitulating the diurnal accumulation profile of hexosylceramides (revised Fig 4A). In our recent publication, we have shown that in non-synchronized islets derived from T2D subjects, most of the core-clock genes were downregulated as compared to ND donors ^5^ (presented here as Fig. 2 Referee 3). At the same time, in the model of clock disruption by siCLOCK ^6^ (Fig. 3 Referee 3), some core-clock transcripts were downregulated (*CLOCK*, *REV-ERBα*, *DBP*, *PER3*), while others were upregulated (*BMAL1*, *CRY1*). Given that both T2D islets and siCLOCK transfected islet cells from ND donors exhibit upregulation of *CERS2* and *UGCG* in our present work, we hypothesize that gene expression of these enzymes might be negatively controlled by one or several core-clock components that are downregulated in both conditions (*CLOCK, REV-ERBα, DBP, PER3*).

Following constructive suggestion by the Referee, we analyzed peaks corresponding to core-clock protein binding on the promoter regions of *CERS2* and *UGCG* based on ChIP-seq dataset available from the ENCODE consortium. The underlying experiments were conducted in human lymphocyte K562 cell line and in liver HepG2 cells. The peak of BHLHE40 protein binding was identified in the intron region of *CERS2,* and in the promoter region of *UGCG*, as depicted in Fig. 4 Referee 3. Further ChIP analyses of BHLHE40 and other core-clock components’ binding to the regulatory sequences of these key lipid biosynthesis enzymes in human pancreatic islet cells would be required to identify the exact molecular interaction between core-clock components and genes encoding for the key enzymes in sphingolipid metabolism.

New qRT-PCR experiments conducted following this insightful suggestion by the Referee were included as new Supplementary Figure 1 (revised Fig S1) into the manuscript.

The following text was added to the Results:

“Rhythmic expression of key core-clock transcripts validated efficient *in vitro* synchronization of human pancreatic islets (Fig S1).”

“Strikingly, the *UGCG* mRNA expression measured around the clock in the islets from ND donors exhibited a rhythmic profile (p = 0.05 as assessed by JTK_Cycle) with the trough around 20-24h following *in vitro* synchronization, corroborating the temporal profile of *BMAL1* transcript (Fig S1), and recapitulating the diurnal accumulation profile of HexCer (Fig 4A).”

**Figure 1 Referee 3. Oscillations of core-clock and clock-related genes in forskolin-synchronized human islets.**

RT-qPCR gene expression profiles of core clock genes BMAL1, CLOCK, PER2, CRY2, REV-ERBα (REVA), and clock output gene DBP normalized to the mean of 9S and HPRT in ND human islets synchronized *in vitro*. The data are expressed as mean ± SEM for n = 4 (n = 3 for CRY2) independent experiments, each using islets from one human donor. Note anti-phasic expression of BMAL1 compared to the profiles of PER2, REV-ERBα, and DBP.

**Figure 2 Referee 3. Pancreatic islets derived from T2D human donors bear disrupted circadian clocks (from** ^5^**).** (A) Altered expression of core-clock genes in non-synchronized human T2D islet cells. The expression levels of *CLOCK* (n = 16 ND; n = 15 T2D donors); *BMAL1* (n = 15 ND; n = 14 T2D donors); *PER1*, *PER2*, *PER3*, *CRY1* (n = 13 ND and T2D donors); *DBP* (n = 11 ND; n = 12 T2D donors); *REV-ERBα* (n = 14 ND and T2D donors); *NFIL3* (n = 8 ND; n = 7 T2D donors); and *CRY2* (n = 10 ND; n = 9 T2D donors) transcripts.

**Figure 3 Referee 3. *CLOCK* knockdown disrupts efficiently molecular clock in the human islet cells (from** ^6^**)** Human islet cells were attached to a laminin-coated surface and transfected with scrambled siRNA (*siControl*) or siRNA targeting *CLOCK* (*siClock*). Cells were harvested 7 days later for qRT-PCR analysis of *CLOCK* (A), *BMAL1*, *CRY1,* *PER3*, *REV-ERBα* and *DBP* transcript levels (B) and normalized to the housekeeping gene *S9*. Data are presented as mean ± SEM of N = 12 experiments, each using islets from one donor.

**Figure 4 Referee 3. Protein binding sites on *UGCG* and *CERS2* based on ChIP-seq dataset delivered by the ENCODE consortium.** *In silico* analysis of ChIP-seq peaks within promoter or regulator elements of *UGCG* and *CERS2* genes based on the publicly available database of ENCODE consortium (<https://www.encodeproject.org/>). BHLHE40 binding sites are highlighted with red frames. For *UGCG*, CACGTG was identified as binding motif. The experiments were performed in K562 (human lymphoblasts) for *UGCG,* and in HepG2 and K562 for *CERS2*.

2. The hypothesis that lipid mis-regulation in T2D and by clock disruption inhibits insulin secretion by increasing membrane rigidity is compelling, however a missing piece of data that would strengthen this argument would be to show that PDMP or myriocin treatment increases the fluorescence GP ratio. However, if the contribution of ceramides and sphingolipids to GSIS occurs independently of membrane dynamics the authors should explain this more thoroughly. It is also surprising that myriocin (ceramide turnover inhibitor) treatment reduces basal insulin secretion while PDMP (UGCG activator) reduces glucose-stimulated insulin secretion. It could be useful to compare effects of myriocin and PDMP on non-metabolic secretagogue-stimulated insulin secretion (such as KCl) to exclude impacts of metabolic and second messenger signaling pathways contributing to the mobilization and exocytosis of insulin vesicles.

We are grateful to the Referee for this excellent suggestion that prompted us to conduct several additional insightful experiments.

During the revision timeframe, we obtained 3 new islet batches from 3 ND human donors (for clinical characteristics, see revised Table 1), that allowed us to conduct KCL-triggered insulin secretion experiments in the presence of myriocin or PDMP on human primary islet cells. The experiments showed a strong KCL-induced inhibition of insulin secretion in the presence of PDMP that reached statistical significance, and a trend to decreased insulin secretion in the presence of myriocin (Revised Fig 6C, E).

Since we were not certain to be able to obtain sufficient number of human islet batches for this experimental series within the revision time span of 1 month, we performed in parallel KCL-stimulation experiments on human islet cell line EndoC-bH1, in 2 independent experimental repetitions. In the presence of PDMP, EndoC-bH1 cells exhibited strong inhibition of insulin secretion following KCL stimulation (Figure 5 Referee 3), in line with the results obtained in human islets (revised Fig 6C, E). We thus decided not to include the experiments with human EndoC-bH1 cells to the revised manuscript, to avoid diluting the main messages of this work that are entirely based on the experiments in human pancreatic islets. It is of course reassuring that the outcome of the experiments addressing the impact of lipid biosynthesis inhibitors on stimulated insulin secretion performed in EndoC-bH1 cells mirror the results obtained in human islets.

In summary, our new experiments in human islets show a strong KCL-induced inhibition of insulin secretion in the presence of PDMP which reached statistical significance, and a trend to decrease insulin secretion in the presence of myriocin (revised Fig 6C, E). These data further suggest that the effect of PDMP is mediated via inhibiting insulin granule exocytosis, rather than by affecting glucose sensitivity.

“The hypothesis that lipid mis-regulation in T2D and by clock disruption inhibits insulin secretion by increasing membrane rigidity is compelling, however a missing piece of data that would strengthen this argument would be to show that PDMP or myriocin treatment increases the fluorescence GP ratio. However, if the contribution of ceramides and sphingolipids to GSIS occurs independently of membrane dynamics the authors should explain this more thoroughly.”

Following this constructive suggestion by the Referee, we assessed the membrane fluidity in human islet cells derived from the same new 3 ND donors described in the previous response. Cultured human islet cells obtained by gentle trypsinization were subjected to 1-h PDMP- or myriocin treatment that was followed by bioimaging using NR12S (new Fig 5C). GP quantification of NR12S fluorescence emission was done in non-treated control islet cells, and in those treated during 1 h with PDMD or myriocin. Significant increase in membrane rigidity was observed after both treatments in comparison to non-treated counterparts (new Fig 5C), suggesting that perturbation of sphingolipid biosynthesis either by inhibition of ceramide synthesis or by inhibition or glucosylceramide synthesis results in increased membrane stiffness. Although both drugs reduced the membrane fluidity to comparable extent, and significantly decreased basal insulin secretion, the effect of PDMP on glucose and KCL-stimulated insulin secretion was much stronger compared to that by myriocin. This difference may stem from a complex interaction of PDMP with the components of insulin secretion pathways, and not solely via changing the membrane fluidity. Indeed, in addition to glucosylceramide synthase inhibition, PDMP was shown to attenuate mTOR signaling pathway, and to induce lysosomal lipid accumulation ^3^. We entirely agree that these effects may interfere with insulin release and glucose sensing, and thus play a role in observed inhibition of insulin secretion. Our new experiments conducted during the revision period indicate that application of PDMP during triggering glucose-independent depolarization of human islet cells with 30 mmol KCL for 1-h inhibits insulin secretion (new Fig 6E). These data suggest that effect of PDMP rather relies on granule exocytosis, and not on insulin sensing. An additional study, that employed direct downregulation of *UGCG* via siRNA in mouse islets that bypasses potential off-target effects of PDMD, also resulted in major inhibition of insulin secretion ^4^, further suggesting specific role of this enzyme in insulin exocytosis.

These important newly added points are now summarized in the Discussion as following:

“In addition to glucosylceramide synthase inhibition, the attenuation of mTOR signalling pathway and lysosomal lipid accumulation reported following PDMP treatment ^76^ may partly account for its inhibitory effect on insulin secretion. Importantly, knock-down of *UGCG* by siRNA in mouse islets also resulted in major insulin secretion defects ^77^, further suggesting that UGCG plays a key role in the observed phenotype.”

**Figure 5 Referee 3.** **Inhibitors of sphingolipid synthesis, PDMP and myriocin, perturb KCL-stimulated insulin secretion by cultured EndoC-bH1 cells.** Inhibitory effects of ceramide analog PDMP (left) and myriocin (right) on KCL-induced (1-h at 30 mmol KCL) insulin secretion in human EndoC-bH1 cells. Data represent values normalized (Ins/residual Ins) to the total residual insulin content and are expressed as mean ± SD for n = 2 repetitions.

**Revised Fig 6. Inhibitors of sphingolipid synthesis, PDMP and myriocin, perturb insulin secretion and circadian oscillations by human pancreatic islets**

Inhibitory effects of myriocin (A-C) and ceramide analog PDMP (D, E) on basal (1-h at 2.8 mmol glucose), glucose-induced (1-h at 16.7 mmol), and KCL-induced (1-h at 30 mmol KCL) insulin secretion in human islet cells in vitro. Data represent values normalized (Ins/residual Ins) to the total residual insulin content (Total residual Ins (mU/L) presented on adjacent graphs) and are expressed as mean ± SEM for n = 7 ND donors (A), n = 3 T2D donors (B), n = 3 ND donors (C), n = 5 ND donors (D), and n = 3 ND donors (E). The difference is tested by paired two-tailed t test; p* < 0.05, p** < 0.01.

See also Figure S4.

(F) Average raw (left) and detrended (right) Per2-luc bioluminescence profiles of human islets in the presence of myriocin or PDMP during the entire bioluminescence recording.

(G) Effect of myriocin and PDMP on principal circadian parameters (phase, period length, and amplitude) of Per2-luc oscillations. Data are represented as mean ± SEM, n = 4 ND donors. The difference is tested by two-way ANOVA test with Bonferroni posttest; p * < 0.05 (as compared to the control counterpart synchronized with forskolin in the absence of these compounds).

**Revised Fig 5. Lipid membrane fluidity of the human pancreatic islet cells is attenuated in T2D islets, in ND islets upon siClock mediated clock disruption, and in ND islets following perturbation of ceramide metabolism by PDMP or myriocin**

Live cell imaging of human islet cells stained with NR12S dye.

(A) Representative ND (left) and T2D (right) human islet cells. The graph on the right summarizes the quantification of fluorescence GP ratio for n = 4 ND and n = 3 T2D donors.

(B) Representative images of ND islet cells transfected with scrambled siRNA (siControl, left) bearing functional clocks and siClock targeting CLOCK protein that bear perturbed oscillators (right). The graph on the right summarizes the quantification of fluorescence GP ratio for n = 3 donors.

(C) Representative images of non-treated control ND islet cells (left), cells following 1-h treatment with PDMP (middle) or myriocin (right). The graph summarizes the quantification of fluorescence GP ratio for n = 3 human donors. Note the significant upregulation of membrane rigidity for T2D islet cells (A), ND cells with compromised clocks (B, siClock), and ND cells with impaired sphingolipid metabolism (C, PDMP and myriocin).

Graph data are represented as mean ± SEM; unpaired two-tailed t test as compared to control, p * < 0.05, p ** < 0.01.

Minor points:

1. The authors note that a number of phospholipid classes displayed non-circadian temporal profiles (p.5), raising the possibility that their synthesis might be related to cAMP induction caused by the forskolin pulse used to synchronize the islets. Do these temporal profiles display an induction early in the time course followed by a steady decline after washout of forskolin?

Indeed, the Reviewer raises a very interesting point. Unfortunately, we did not perform lipidomic analyses on the islets collected sooner than 12 h following the synchronization by forskolin to exclude the confounding effect of early response to *in vitro* synchronization. Such approach is widely accepted in the field, with certain groups starting the collection even later for that same reason (36 h following synchronization in ^7^). We believe that although being an interesting question *per se*, the immediate early effect of cAMP on the lipid metabolism requires a different experimental setup, and indeed represents an important question for the follow-up studies.

2. T2D islets were assayed at 12 and 24 hours after forskolin pulse (p. 8) but a rationale for these time points was not chosen. Please state why these times were selected.

We thank the Referee for this pertinent and constructive criticism. We entirely agree that experimental design based on 2 time points 12 h apart from each other does not allow recapitulating the lipid rhythmicity, in particular for the metabolites that do not peak at CT12/24. Two time points instead of usual 7 was a compromise that had to be done due to the lack of T2D islet material that is even more important than for ND islets (see the response to previous comment 2). Based on our available sources of human islets, we simply cannot obtain enough IEQ from T2D donors for the full-blown around-the-clock lipidomic design as was done for the experiments in ND islets presented in Figure 1. It is clear that any 2-time point design would lead to missing part of the rhythmic lipid metabolites, and all metabolites that peak at CT6/18 will not be qualified as changing across 24 h in our study design.

The opposite time-points 12 h and 24 h of the oscillatory cycle were chosen based on the transcriptional data, and on the oscillatory profiles of most of the lipids in ND donors. Indeed, these 2 points correspond to peak and trough of the core-clock gene *BMAL1* expression level (revised Fig S1), and of the rhythmic profiles obtained for lipid species in most of the examined ND donors (4 out of 6 donors, Fig 1C).

Following constructive criticism by the Referee, we underscored this important limitation of our study design in T2D islets in the following remarks to the revised text of Results and Discussion:

Results

“The selected time-points correspond to peak and trough of the core-clock gene *BMAL1* expression level (Fig S1), and of the rhythmic profiles obtained for lipid species in most of the examined ND donors (4 out of 6 donors, Fig 1C).”

Discussion

“Due to the limited availability of human islets derived from T2D donors, we were unable to perform complete around the clock experiments for this part and compromised on 2-timepoint design. As a result, the temporal changes in lipid metabolites that are peaking at CT6 and CT18 were likely missed in T2D islets.”

Furthermore, we toned down the message on temporal analyses in T2D islet in the respective sub-chapter title in Results, since it is indeed an overstatement, and reformulated it as following:

“Lipidomic profiling reveals major changes in lipid metabolites at 2 time points in human T2D pancreatic islets synchronized *in vitro”*

3. In figure S4 PDMP further down-regulates GSIS in islets from a T2D donor. The authors should explain why this might be occurring since a hypothesis is that increased UGCG activity underlies circadian and diabetes-induced secretory impairment. Is the extent of suppression similar or different to ND islets and does PDMP suppress GSIS in siCLOCK islets?

We thank the Referee for raising this fair criticism. Indeed, the upregulation of *UGCG* transcript expression observed in T2D and ND islet cells transfected with siCLOCK does not corroborate in a straight-forward manner the results stemming from glucose- and KCL-induced insulin secretion in both T2D and ND islets in the presence of UGCG inhibitor PDMP. Our current hypothesis is that overexpression of *UGCG* in T2D islets, and in ND islets transfected with siCLOCK, represents a compensatory mechanism that cells may utilize to reduce the accumulation of toxic ceramides by shunting them toward glucosylceramides. Inhibition of this pathway by PDMP would result in accumulation of ceramides and/or activation of alternative pathways to reduce the intracellular ceramides concentration, thus resulting in perturbation of additional mechanisms involved in insulin secretion. Hence, we may consider the effect of PDMP inhibition as a complex perturbation of ceramide synthesis pathways, rather than as a simple reduction of UGCG function.

4. In figure 6 panel A it appears the data are mislabeled. The colors for myriocin and PDMP are switched for the left and right panels.

We thank the Reviewer for pointing out this unfortunate error. We corrected the Figure legend of the panel F to render it consistent with the legend of the panel G (see Revised Fig 6).

5. Since human islets contain a relatively higher proportion of glucagon secreting alpha cells and the methods employed here do not appear to discriminate between alpha and beta cells the authors should comment on the possibility that circadian-induced changes in lipid metabolism and membrane dynamics occur in alpha cells impacting glucagon secretion. Since they have previously generated circadian RNA-seq data (in mouse) in alpha and beta cells it would be useful to know if key lipid metabolism genes are differentially controlled by the clock in these cell populations.

Following this pertinent suggestion by the Referee, we looked at our previously published database of daily transcriptomic profiles in FACS-separated mouse alpha- and beta-cells (Supplemental Dataset 1 in ^8^). In mouse islets, *Ugcg*, *Cers2*, *Cept1*, *Chka* are equally expressed in alpha and in beta cells. Unlike the observation in *in vitro* synchronized human islet cells (revised Fig 4), the temporal expression profiles of *Ugcg* and *Cers2* in mouse islet cells collected around-the-clock were considered non-rhythmic in both alpha- and beta- cells. The profile of *Chka* expression was qualified rhythmic in both cell types with similar rhythmic parameters, while the rhythmicity of *Cept1* expression was non-conclusive. These differences between the observed temporal expression profiles could be attributed to the specie-specificity, and to a different methodological setup, with *in vivo* study conducted in mice in our previous work versus *in vitro* synchronization in human islet cells.

6. In figure 4A only two cells can be seen in the field for each condition. If possible the authors should choose a more convincing image.

Following this pertinent criticism, we replaced the original images in revised Fig 5A (former Fig. 4A) with those comprising higher number of cells in the field of represented images.

7. Figure S3B shows pathways with p values typically higher than what is traditionally used in gene ontology. The case for these pathways being overrepresented could be strengthened by listing the number of genes represented in each of these pathways overlapping with DEGs in siCLOCK islets.

We are grateful to the Referee for raising this point. Indeed, in Fig S3B, we arbitrary chose the p-value cutoff at 0.1 to cover first 9 pathways enriched in upregulated genes. Since the main focus of this panel was on sphingolipid pathway (p = 0.0575), we now listed all three significantly upregulated genes from this pathway in the Figure legend.

Following constructive criticism by the Referee, we introduced the following changes into revised text of the Results chapter:

“Moreover, KEGG pathway enrichment analysis of all significantly upregulated transcripts in clock-compromised islets revealed activation of sphingolipid metabolism pathway (p = 0.0575; Fig S3B).”

List of references

1	Chua, E. C. *et al.* Extensive diversity in circadian regulation of plasma lipids and evidence for different circadian metabolic phenotypes in humans. *Proceedings of the National Academy of Sciences of the United States of America* **110**, 14468-14473, doi:10.1073/pnas.1222647110 (2013).

2	Loizides-Mangold, U. *et al.* Lipidomics reveals diurnal lipid oscillations in human skeletal muscle persisting in cellular myotubes cultured in vitro. *Proceedings of the National Academy of Sciences of the United States of America* **114**, E8565-E8574, doi:10.1073/pnas.1705821114 (2017).

3	Hartwig, P. & Hoglinger, D. The Glucosylceramide Synthase Inhibitor PDMP Causes Lysosomal Lipid Accumulation and mTOR Inactivation. *Int J Mol Sci* **22**, doi:10.3390/ijms22137065 (2021).

4	Jennemann, R. *et al.* Gangliosides modulate insulin secretion by pancreatic beta cells under glucose stress. *Glycobiology* **30**, 722-734, doi:10.1093/glycob/cwaa022 (2020).

5	Petrenko, V. *et al.* In pancreatic islets from type 2 diabetes patients, the dampened circadian oscillators lead to reduced insulin and glucagon exocytosis. *Proc Natl Acad Sci U S A* **117**, 2484-2495, doi:10.1073/pnas.1916539117 (2020).

6	Saini, C. *et al.* A functional circadian clock is required for proper insulin secretion by human pancreatic islet cells. *Diabetes Obes Metab* **18**, 355-365, doi:10.1111/dom.12616 (2016).

7	Perelis, M. *et al.* Pancreatic beta cell enhancers regulate rhythmic transcription of genes controlling insulin secretion. *Science* **350**, aac4250, doi:10.1126/science.aac4250 (2015).

8	Petrenko, V. *et al.* Pancreatic alpha- and beta-cellular clocks have distinct molecular properties and impact on islet hormone secretion and gene expression. *Genes Dev* **31**, 383-398, doi:10.1101/gad.290379.116 (2017).
